# Memorability-based multimedia analytics for robotic interestingness prediction system using trimmed Q-learning algorithm

**DOI:** 10.1038/s41598-023-44553-1

**Published:** 2023-11-13

**Authors:** Hasnain Ali, Syed Omer Gilani, Asim Waris, Umer Hameed Shah, Muazzam A. Khan Khattak, Muhammad Jawad Khan, Namra Afzal

**Affiliations:** 1 School of Mechanical & Manufacturing Engineering, National University of Sciences and Technology, Robotics & AI, Islamabad, 44000 Pakistan; 2 Department of Electrical, Computer, and Biomedical Engineering, Abu Dhabi University, Abu Dhabi, UAE; 3 School of Mechanical & Manufacturing Engineering, National University of Sciences and Technology, Biomedical Engineering & Sciences, Islamabad, 44000 Pakistan; 4 Department of Mechanical Engineering and Artificial Intelligence Research Center, College of Engineering and Information Technology, Ajman University, Ajman, UAE; 5 Department of Computing, Quaid i Azam University, Islamabad, 44000 Pakistan; 6 Department of Biomedical Engineering, University of Engineering and Technology, Lahore, 54000 Pakistan

**Keywords:** Computational science, Computer science, Information technology, Software

## Abstract

Mobile robots are increasingly employed in today’s environment. Perceiving the environment to perform a task plays a major role in the robots. The service robots are wisely employed in the fully (or) partially known user’s environment. The exploration and exploitation of the unknown environment is a tedious task. This paper introduces a novel Trimmed Q-learning algorithm to predict interesting scenes via efficient memorability-oriented robotic behavioral scene activity training. The training process involves three stages: online learning and short-term and long-term learning modules. It is helpful for autonomous exploration and making wiser decisions about the environment. A simplified three-stage learning framework is introduced to train and predict interesting scenes using memorability. A proficient visual memory schema (VMS) is designed to tune the learning parameters. A role-based profile arrangement is made to explore the unknown environment for a long-term learning process. The online and short-term learning frameworks are designed using a novel Trimmed Q-learning algorithm. The underestimated bias in robotic actions must be minimized by introducing a refined set of practical candidate actions. Finally, the recalling ability of each learning module is estimated to predict the interesting scenes. Experiments conducted on public datasets, SubT, and SUN databases demonstrate the proposed technique’s efficacy. The proposed framework has yielded better memorability scores in short-term and online learning at 72.84% and in long-term learning at 68.63%.

## Introduction

The simulation of the unknown environment is a tedious task in mobile robotics^1^. The role of route planning and executing the robots on the trajectories is done using a map model. This is known as ‘exploration.’ Designing an intelligent exploration model is one of the developing real-world robotic applications^2^. The human brain can effortlessly perceive objects in the visual environment. It takes only a few milliseconds^3^ to differentiate the objects presented in the environment. Indeed, training artificial systems that equalize human-level performance to differentiate the objects in an image is still challenging^4–7^. Performing the navigation task is a challenging task because of the available information. The representation of the environment is required to perform navigation tasks. To design an autonomous system, the representation of the information related to the initial and intended position is also significant.

Scene recognition (SR) is a rapidly growing domain that received much attention in recent past years. It is a tedious task that looks for better methods to classify the objects at an appropriate time. It is one of the vital processes for the design of robots’ navigation and exploration^8^. The recognition of interesting scenes plays a vital role in the development of intelligent exploration. It is also one of the fundamental abilities of mobile robots. It helps to make better decisions for the robot navigation task. Consider finding the door with a hole in the wall, which could affect forecasting the next desired point. Regardless of it, the existing methods are difficult in unknown environments. In this case, the robots find engaging scenes and some repetitive scenes that impact the robot exploration process. There is a chance of losing interest in the interesting scenes. The current approaches, such as interestingness detection, saliency detection, anomaly detection, novelty detection, and meaningfulness detection, can’t learn the scenes in both offline and online schemes^9^.

To gain a complete understanding of images, the precise estimation and analysis of the locations and concepts of the scenes in each image is an important task. This is referred to as ‘object detection’ which provides valuable information to perceive the semantic concepts of images. The design of learning systems has a great impact on object detection techniques. It is a tedious and time-consuming due to issues such as deviation in viewpoints, poses, occlusion, and lighting conditions. It receives much attention to determine the objects presented in the given image and their relevant classes. Therefore, the processes involved in object detection are^10^:

- Selecting the informative region: The presence of different objects in an image will have different aspect ratios. The analysis of multi-scale sliding windows helps to perceive the whole image. However, it has many pitfalls in locating the exact position of an object (or) scene. The chance of irrelevant regions may be analyzed, which leads to computationally expensive.
- Extracting the significant features: The extraction of visual features facilitates the semantic and robust representation. The diverse nature of images, like faded appearance, illumination, and backgrounds, will deteriorate the design of the feature descriptor.
- Classification: Finally, a classifier is required to differentiate the objects from their categories. This representation makes it more hierarchical, semantic, and informative for predictions.

The main contributions of the paper are:

- To address the unknown environment, a novel Trimmed Q-learning algorithm that leverages the hyperparameters of short-term, long-term, and online learning modules.
- Inspired by the lateral placement (LP) strategy, the interesting scenes are trained via candidate roles.
- A Novel Trimmed Q-learning algorithm is designed to improve maximizing the expected action value. Long-term, short-term, and online learning training must be efficient regarding scene recall ability.

The paper is organized as follows:

- Section “Related work” presents the ‘Related surveys’ that discuss the scope of the existing studies.
- Section “Proposed framework” presents the ‘Proposed framework’ that discusses the working module of the memorability-based interestingness prediction system.
- Section “Experimental results and discussion” presents the ‘Experimental results and discussion’ that portrays the evaluation of the designed framework.
- Section “Conclusion” presents the ‘Conclusion’ that discusses the study’s findings.

## Related work

The concept of vision-based robot exploration has been a vital goal in the robotics research field. It remains a challenging task for robots equipped with vision sensors. The robotic navigation is adjourned using model-based and appearance-based approaches to detect interesting scenes. Model-based approaches portray the derivation of knowledge using the 3D model. With the help of sensor data, the localization of the scene is estimated using global and local models. The features such as lines^11^, planes^12^, and points^13^. In the case of unknown model exploration, the learning step is involved. The human operator controls the robot’s actions, wherein the reconstruction of that performance is handled by hierarchical bundle adjustment. In the line case, the odometry^14^ is integrated with the visual tracking system to derive the feature coordinates. Several studies have defined the concept of an autonomous mapping model using Simultaneous Location and Mapping (SLAM). These approaches help to discover the new region but do not achieve the intended location. Finding the present position is a tedious task. In^15^, at the learning step, the navigation is estimated from the combination of different features obtained from the image trajectories mapping module. The analogy of visual complexity has been studied using Shannon entropy^16^. The analysis of complex images includes more redundant information by estimating the entropy. The entropy-based measures are employed to operationalize the visual clutter^17^. Relied on the entropy value, the images are cluttered and disorganized.

The appearance-based approach is referred to as the topological approach. It does not take the need for a 3D model. It performs on the available sensor space and is represented by a topological graph. Herein, the node denotes the description of the current position, and the link represents the connection between nodes for navigation purposes. The images are acquired and analyzed in the learning step. The concept of localization is employed to compute the likelihood score between ground truth and different images. The global descriptors^18,19^ can also be used for estimating the likelihood. Similarly, color histograms^20^ and image gradients^21,22^ were also used to analyze the entire image. Pertaining to it, the localization of robots using local descriptors is studied using photometric invariants^23^ and SIFT points^24^. Several techniques have been introduced to assist mobile robots during navigation^25^. A unique motion feature is selected from each image^26^. Robots perform the next navigation step based on the closest view of an image. However, it could not handle the deviation when it’s been away from the planned path. The robots are converged using a visual servings loop^27^ that could measure the error and achieve the intended positions. Sometimes, the convergence towards the intermediary position fails to reach the intended position.

The role of the interestingness measure was introduced in later years. It is a kind of subjective measure that looks for annotated features. To characterize the judgments, the association between human visual interestingness and image features is studied^28^. It is keenly observed in the supervised learning methods specific to training modules. In^29^, the three features, such as composition, content, and illumination are used to measure the interestingness of the image. In^30^, the social media platforms such as YouTube and Flickr are used to evaluate the interestingness from image to video using visual features. The main cause of the interestingness measure is evolving as a unified learning model. It is made to recognize the outliers from human annotation^31,32^. Deep learning has been adopted for forecasting the interestingness measures. In^33^, a modulated support vector regression is introduced on the animated GIF inputs. Then, a customized CNN is designed to recognize the salient and non-salient sliding window frames using video inputs. A combination of two deep-ranking networks^34^ was studied to enhance the performance of the interestingness measure. Similarly, in^35^, the CNN and LSTM are combined to extract the learning features for media inputs.

The human annotation used for training is computationally expensive^36^. Concurrently, it is studied in unsupervised learning modules^37^. The density ratio algorithm with the HOG features^38^ is studied. However, it is not suitable for adaptive constraints. In^39^, the autoencoder technique is employed as an unsupervised learning step for better feature extraction. In some scenarios, an autoencoder is employed to find the regularities in long-term videos^40^. The dropout layer in the autoencoder is analyzed under pixel-wise saliency detection^41^. It is extended using a spatiotemporal autoencoder^42^ that extracts spatial and temporal features. Many researchers have predicted visual complexity using information theoretics under different human perceptions^43^. Multiple complexity scores are evaluated for different perceptions of the image. Though it has given better accuracy, the online learning process is still low. The most recent work focuses on developing neural models of perceptual image complexity, finding that visual complexity information arises within the feature maps of deep convolutional networks^44^ and, similarly, that multiple regions across the brain are involved with the representation of the complexity inherent in naturalistic stimuli^45^. In^46^, Rewards based learning process was focused to innovate in episodic memory. It was explored on the “couch-potato” issues of prior work. The deployed agents has established the instant self-actions. It was tested in visually rich 3D environments in ViZDoom, DMLab and MuJoCo. A learning adaptive based imagination approach^47^ was studied to enhance the reliability of the formed dynamics models. It was explored on the latent space and the intrinsic rewards of the learning process. Dual system based motor learning model^48^ was studied to arbitrate the meta-controller between model based and model-free decisions. In specific to, the reliability of the learned models was explored from the intrinsic feedback signals. The results show that our approach outperforms the compared methods and learns near-optimal grasping policies in dense- and sparse-reward environments. A general end-to-end diversity-augmented intrinsic motivation for deep reinforcement learning which encourages the agent to explore new states and automatically provides denser rewards was studied^49^. It was explored in MuJoCo, the approach improves on prior techniques for tasks using the standard reward setting, and achieves the state-of-the-art performance on 12 out of 15 tasks containing delayed rewards. Plan2Explore, a self-supervised reinforcement learning agent^50^ that tackles both these challenges through a new approach to self-supervised exploration and fast adaptation to new tasks, which need not be known during exploration.From the conducted survey, the concept of Reinforcement Learning (RL) in interesting scene measure has been studied to provide a set of sophisticated tools for learning robotic controls. It works on the dynamic variables of state and actions in robotic field.

## Proposed framework

The derivation of required information from a scene is not limited to the practical environment. Depending on the application, the prediction of scenes from an image might vary, i.e. prediction of all presented objects in a scene, prediction of organized objects, prediction of similar objects, prediction of interesting scenes in an object, and so on. These complex information systems are maintained by visual queries, called as visual systems. If the represented objects are not linked to the scene, then understanding the scene with visual memory will be helpful. The design of complex visual queries assists in binding the objects represented under retainable memory. The objects are learned from both online and offline modes. Online learning schemes are quite low in dealing with complex visual queries compared to offline schemes.

### System model

Consider a set of images representing the different scenes. It is presented in the matrix, I = [${s}_{1},{s}_{2},{s}_{3},{s}_{4}\dots .{s}_{c}]$ $\in$ ${R}^{F\times T}$. The required visual memory schema (VMS) is represented as M = [${m}_{1},{m}_{2},{m}_{3},{m}_{4}\dots .{m}_{c}] \in$ ${R}^{T}$, where ${v}_{i }\in {R}^{F}$ and F is the dimension of features; R is the real number field; T is the set of training videos and ${v}_{i}$ is the set of variables. The objects and the regions of the scene are presented in visual schemas that include physical and spatial properties. Specifically, the different regions of a scene with required information are associated, sorted, and encoded into a visual memory. It is then retrieved with the efficient memory schema. However, the VMS can bring deviated interference between observed and predicted information. Each image’s visual region map is formulated and remembered for further use. It encodes semantic knowledge and episodic memory of an image. Therefore, the VMS may correspond to true and false image memorability. Thus, updating the VMSs according to the online and offline modes is presented.

### Feature extraction

The three main features, annotation, object, and scene of an image are employed to build an efficient VMS. As presented in a dataset, the human annotation varies; thus, normal and difficult annotations are used.

### Long-term learning

The chance that interesting scenes and redundant scenes might become uninteresting scenes during the long-term learning process. Role-based lateral placement (RBLP) is a novel behaviour-based and unsupervised method used in this study. It performs to identify the intra- image associations. The different scenes of an image are communicated over time which are arranged into a learnable set of roles. Once after extracting the features, these are fed into the LP process of the RBLP technique. Lateral placement (LP) is the closest lateral position of a robot from the edge of the pavement when the robot is in motion. A reference line is maintained to eliminate the collapse of the robot during motion. The performance time of each robot is captured from the video frames. Based on that, the distance and speed of the robot are estimated. The estimated object attributes from a frame are considered for defining the candidate roles. The design of role-based learning models relies on the subject of the input frame.

Let’s consider that the ground truth role of each video is unknown and inferred from the connection between annotation and scene attributes. A general system classification method based on the interesting scenes labelled as ‘normal and difficult’ is used for defining the functional roles. Each video is labelled as ‘normal’ dealing with a single subject of interesting scenes and ‘difficult’ dealing with multiple subjects of interesting scenes over a period of time. These two labels summarize how a particular video acts as normal and difficult. A video belonging to the same group is considered to have the same role. In some scenarios, the roles are not easily defined due to the descriptive labels. Relying on the external databases, the quality and the count of groups will vary, which might have two different roles with different subjects.

(A) Creating a ‘normal’ profile: To create a normal profile, a data source is maintained to record the linked information involving the frame. Relevant data attributes and scene attributes are used.
(B) Creating a ‘difficult’ profile: To create a difficult profile, a source of data is communicated with the inter-associated frames gathered.
(C) Recognizing the roles: To begin this, all frames are observed over some time. Depending on the LP moves, each video is profiled using the above methods. Similar profiles are grouped and termed as ‘roles’.

### Short-term learning

Initially, the mission of the robot system is started with uninteresting scenes. The set of interesting objects must be studied in the short term to learn the interesting scenes. Henceforth, a supervised object detector is employed in the prior unsupervised model. It is trained in the incremental process to learn in a stipulated period. Hence, a novel Trimmed_Q-learning algorithm is proposed. Q-learning is a kind of reinforcement learning that eliminates the robot’s computational effort and increases its abilities. Since it’s behaviour-based, an improvement is made using reward agents and requires little supervision. The Q-learning algorithm uses Q-tables, which reduces the longer training time. The function approximations of the Q-learning are devised in this study. The proposed Trimmed_Q-learning technique combines the baselines of traditional Q-learning with an improvement in maximising the expected action value. In short-term learning, the underestimated bias in robotic actions must be minimized by introducing a refined set of practical candidate actions. It includes two sets: a set of fitted candidates’ actions with high action values and a set of estimators.

Consider a robotic video interacting with an environment ℇ. The state s of a robot is a high-dimensional vector including s € S, where S is the set of available states. According to the environment, the robot takes the actions a € A = {a^1^, a^2^ …a^n^}, n € N, Number of possible actions. The state transition probability distribution of a robot with an environment is expressed as:

1 $$Prob=S \times A\times S \to {\varvec{R}}{\varvec{o}}{\varvec{b}}{\varvec{o}}{\varvec{t}}.$$

Reward agent R is expressed as:

2 $${\varvec{R}}:S \times A \to {\varvec{R}}{\varvec{o}}{\varvec{b}}{\varvec{o}}{\varvec{t}}\boldsymbol{ }\boldsymbol{ };\mathrm{ With \,\,a \,\,discount \,\,factor} \;\;\boldsymbol{ }\gamma \epsilon [\mathrm{0,1}]$$

The agent gains a new reward information r^t^ for a given time step t and the present state s^t^ ϵ S. Then, a new state s^t+1^ $\epsilon$ S will be generated for the current action a^t^ $\epsilon A$. Therefore, the intention of the agent is to maximize the aggregated rewards by ensuring the fittest policy $\pi :State s \times Action A\to [\mathrm{0,1}]$. In the conventional Markov Decision Process (MDP), the functional value for an action is presented as:

3 $${Q}^{policy \pi } \left({\varvec{s}},{\varvec{a}}\right)={Expect}^{policy \pi } \left [{\sum }_{t=0}^{\infty }{\gamma }^{t}{r}^{t} \vdots {s}_{0}=S; {a}_{0 }=A \right].$$

The fittest policy is obtained by equalizing the Bellman Optimality (BO) constraints which are expressed as:

4 $${Q}^{**}\left({\varvec{s}},\boldsymbol{ }{\varvec{a}}\right)= {Expect}^{\pi **}\sim Prob \left(\gamma \vdots {\varvec{s}},{\varvec{a}}\right)[ Reward R \left({\varvec{s}},{\varvec{a}}\right)+{\mathrm{max}{Q}^{**}}_{a^\prime \epsilon A} \left({\varvec{s}}^\prime,{\varvec{a}}^\prime\right).$$

To approximate the random variables for the short-term learning process, an optimal functional value for an action ${Q}^{*}\left({\varvec{s}},\boldsymbol{ }{\varvec{a}}\right)$ will iteratively increase. Thus, the updated action value is expressed as:

5 $$Q\left({s}^{t},{a}^{t}\right) \to Present \left(Q\left({s}^{t},{a}^{t}\right)\right)+ \propto \left({target y}_{t}^{Q}-Q\left({s}^{t},{a}^{t}\right)\right),$$

where the target value is estimated as:

6 $${target y}_{t}^{Q}={reward}^{t}+ \gamma {Q}_{a^\prime\in A}^{max}\left({s}^{t+1}, a^\prime\right).$$

The trimmed_Q-Learning relives the overestimation bias in frame parameters by maintaining two Q-functions, Q^P^ and Q^U^. One of the Q-learning functions is randomly updated with the target values. It is expressed as:

7 $${target y}_{t}^{Q^\prime}= {r}^{t}+ \gamma {Q}^{U}({s}^{t+1 },\mathrm{arg}\begin{array}{c}max\\ a^\prime\upepsilon A\end{array} {Q}^{P} \left({s}^{t+1}, a^\prime\right).$$

The interesting scenes are trimmed by maximum action value from one of the Q-learnings, which is updated and expressed as:

8 $${target y}_{t}^{tri{m}_{Q}}= {r}^{t}+ \gamma \mathrm{min}\{{Q}^{P}\left({s}^{t+1}, {a}^{*}\right); {Q}^{U}\left({s}^{t+1}, {a}^{*}\right)\}.$$

Finally, the maximum function value is fitted according to the short-term learning parameters. Furthermore, the reward function ($Reward R \left({\varvec{s}},{\varvec{a}}\right)$) is formulated from the expected reward and the observed reward which is obtained from the optimal value of the next state. All reward functions are considered as bounded. The behaviors such as up, down, top and bottom robotic motions are used to estimate the reward function.

### Online learning

Online learning looks for an update within a stipulated period of time. Hence, it combines with the short-term learning module. It demands continuous action control, and so an action-critic framework is formulated. An actor-network $\vartheta (s,\theta )$ and two critic-networks, Q(s, a| $\theta$_1_) and Q(s, a| $\theta$_2_). According to the robotic learning environment, the critic networks are updated using the formula.

9 $${\theta }^{k}\to {\theta }^{k}+ \alpha {\nabla }_{{\theta }_{k}}Expect \left[{\left(Q\left({s}_{t},{a}_{t}; {\theta }^{k}\right)-{target y}_{t}^{Onlin{e}_{tri{m}_{Q}}}\right)}^{2}\right].$$

The target value ${target y}_{t}^{Onlin{e}_{tri{m}_{Q}}}$ is defined as:

10 $${target y}_{t}^{Onlin{e}_{tri{m}_{Q}}}= {r}^{t}+ \gamma \begin{array}{c}min\\ k=\mathrm{1,2}\end{array} \{Q\left({s}^{t+1}, \mu ( {s}^{t+1} ; {\varphi }^{-}\right);{\theta }_{k}^{-} \} ,$$

where ${\varphi }^{-}$ and ${\theta }_{k}^{-}$ are the online hyperparameters of $\varphi$ and ${\theta }^{k}$.

Finally, the fittest policy for an actor $\mu (s; \varphi )$ is updated as follows:

11 $${\nabla }_{\varnothing }=Expect \left[{\nabla }_{a}Q\left({s}^{t}, a ; {\theta }_{1}\right)\right| {\nabla }_{\varnothing }\mu \left({s}^{t};\varnothing \right).$$

The trimming operation removes the underestimation issue during the parameters learning process.

### Memorability-based interesting scene prediction

**Figure 1:**
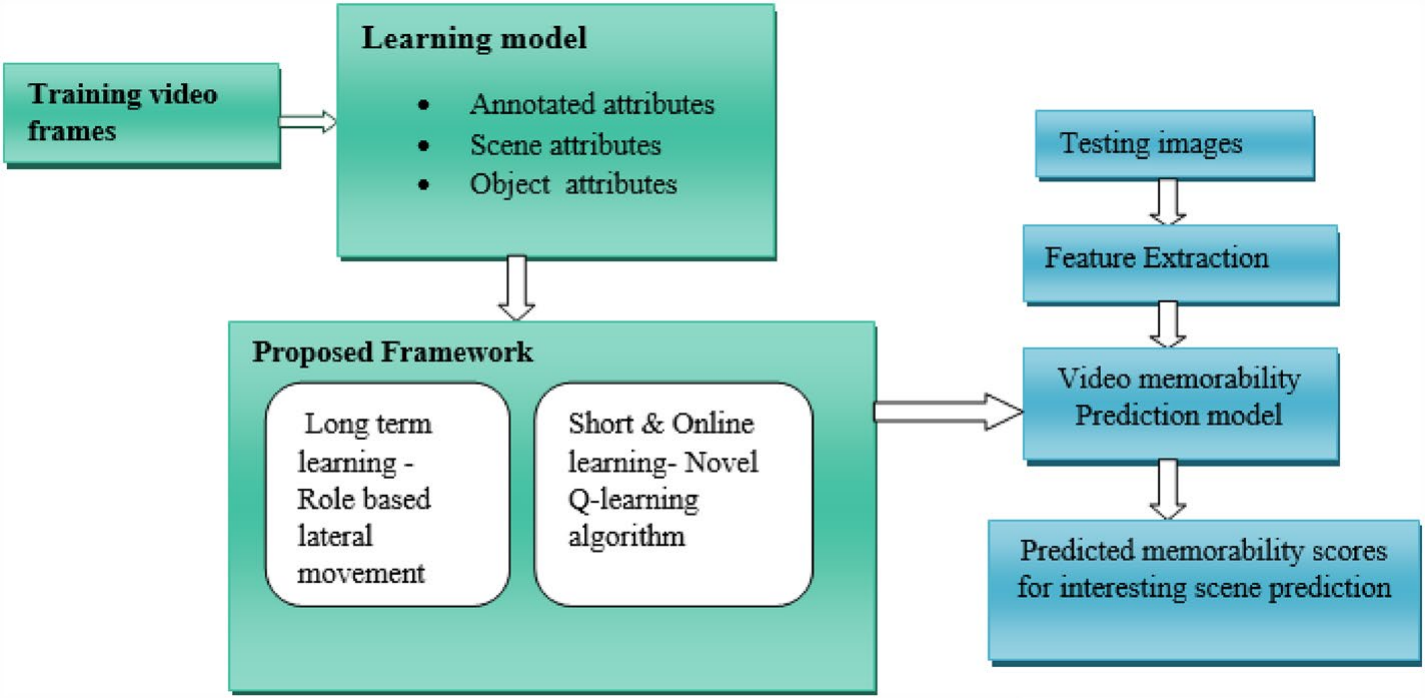
Proposed workflow.

The interesting scene prediction comes from the proper training process as shown in Fig. 1. Hence, the count of candidate action sets is vital in predicting the interesting scenes. The training process of long-term, short-term and online learning must be efficient regarding scene recall ability. In the reinforcement learning module, the agent looks for a good set of candidate actions C that speeds up the target region’s process. Since the robot’s actions are continuous and discrete by nature, the short-term ($${target y}_{t}^{tri{m}_{Q}}$$) and online learning ($${target y}_{t}^{Onlin{e}_{tri{m}_{Q}}})$$ estimators are combined.

In the Q-tables, the Q-function preserves Q^P^ and Q^U^. The learning process’s actions occur on those Q-functions and the experience. Depending on the experience, the Q-functions are updated. Pertaining to it, the Q^P^ is updated as:

12 $${\aleph }^{C}=\left\{j \right|{Q}^{U}\left(s^\prime,{a}_{j}\right) \epsilon \,\,top\,\, C \,\,values \;\; in \;\;{Q}^{U }(s^\prime, .)\}.$$

For Q^P^, the maximum action value a_c_^*^ is estimated from ${\aleph }^{C}$ at state s′. Then, it’s updated as:

13 $${target y}_{t}^{tri{m}_{Q}}=r+ \gamma \mathrm{min}\{{Q}^{U}(s^{\prime}, {a}_{c}^{*}), {max}_{a} {Q}^{P} (s^{\prime}, a)\}.$$

The actions Q^P^ and Q^U^ are explored via a greedy exploration strategy. It balances the overestimation and underestimation bias. At last, it converges to the fittest policy under finite MDP constraints.

## Experimental results and discussion

**Figure 2:**
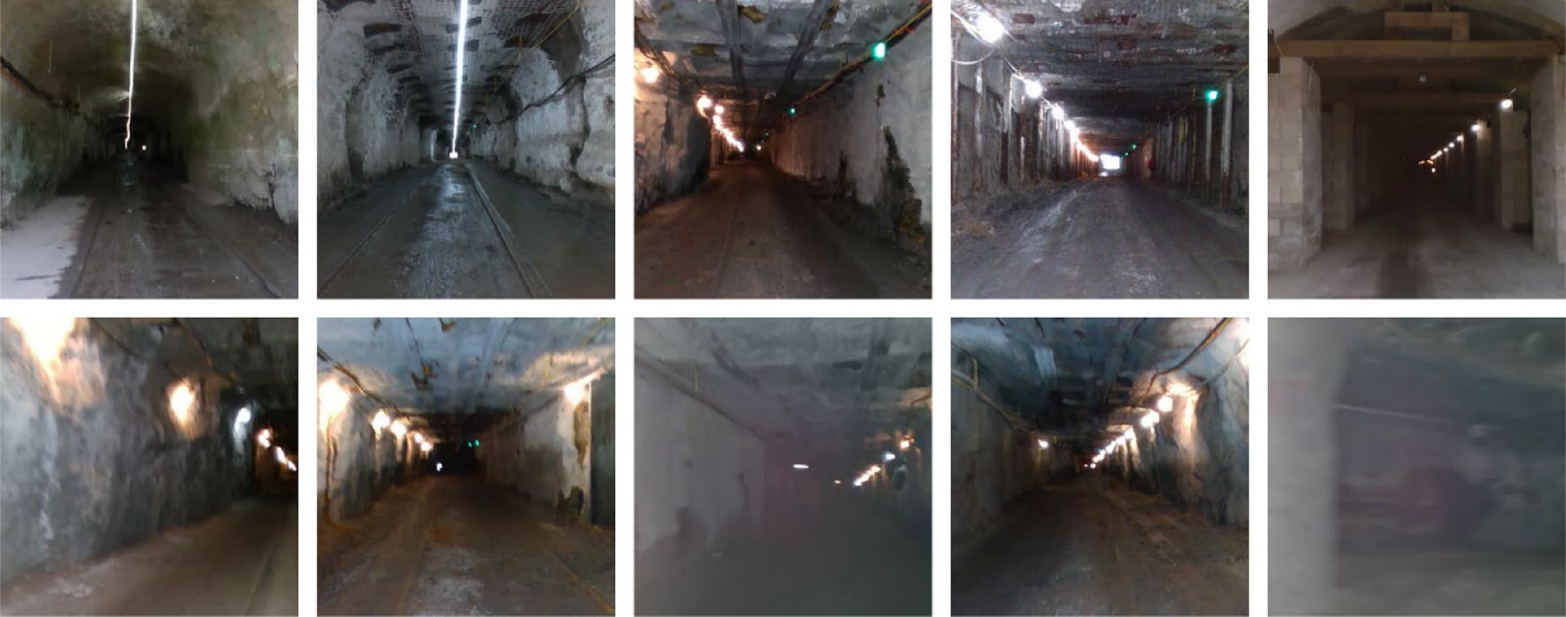
Uninteresting scenes–samples.

**Figure 3:**
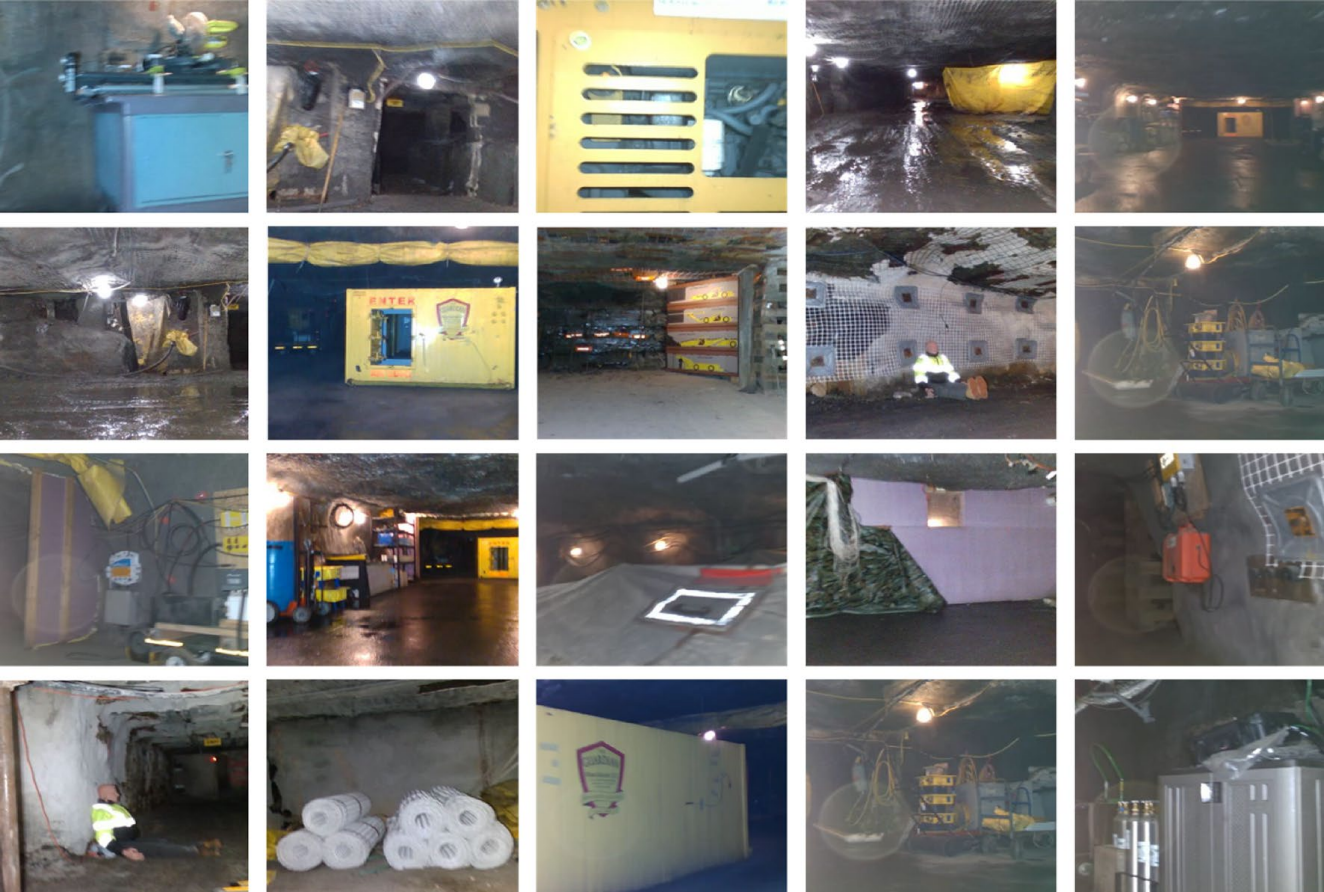
Interesting scenes–samples.

The Subterranean SubT dataset^51^ is employed for the experimental setup and simulation purposes. The dataset is collected by the team of DARPA communities that assists the robots to intelligently explore and exploit in the subterranean environment. This dataset poses many challenges to robots in terms of lighting, incapability of GPS, water dripping and so on. This dataset introduced by defense advanced research projects agency (DARPA) that discusses the underground operations. It explores new approaches rapidly map, navigate, search, and exploit complex underground environments such as human-made tunnel systems, urban underground, and natural cave networks. Therefore, the prediction of the interesting scene is a tedious task. Each video runs from 50 to 85 min and is being annotated as normal and difficult. The Figs. 2 and 3 present the difference between uninteresting and interesting scenes. The proposed framework experiments on these input videos. The learning process is implemented in MATLAB 2019A.

The efficacy of the proposed learning framework is evaluated using the performance metrics such as Precision, recall, F-measure and memorability score. The Table 1 presents the collective performance values of the proposed technique applied on the considered dataset.

**Table 1:** Performance values of each dataset.

Testing data	No. of frames	Selected frames (interesting scenes)	Object & scenes category	Precision	Recall	F1-measure
817-UGV0-Tunnel0	3312	2444	Objects	80.14	78.23	80.12
817-UGV0-Tunnel1	5023	3245	Humans	79.23	80.14	78.45
818-UGV0-Tunnel1	4845	2364	objects	80.00	78.14	79.63
820-UGV0-Tunnel1	8145	5478	Outdoor	80.45	80.17	81.12
821-UGV0-Tunnel1	5255	3124	Outdoor	81.47	80.17	81.23

### Precision

The intention of the precision metric is to enhance the success rate of a predictor system. It is a statistical measure used for validating the predicted interesting scenes to the total count scenes in videos with reference to ground truth information. It is expressed as follows:

14 $$Precision= \frac{count\,\, of \,\,correctly \,\,predicted \,\,interesting\,\, scenes }{total \,\,count\,\, of \,\,interesting \,\,and \,\,uninteresting \,\,video \,\,scenes }.$$

### Recall

The recall is the statistical measure for defining the ability of a developed prediction system. It evaluates the testing videos. It is expressed as:

15 $$Recall=\frac{{\sum }_{j=1}^{TS}({s}_{j}-\overline{s })({g}_{j}-\overline{g })}{\sqrt{{{\sum }_{j=1}^{TS}({s}_{j}-\overline{s })}^{2} } \sqrt{{{\sum }_{j=1}^{TS}({g}_{j}-\overline{g })}^{2}}},$$

where TS is the aggregate count of test video scenes, ${g}_{j}$ is the ground truth value of jth scenes, $\overline{g }$ is the mean ground truth value; ${s}_{j}$ is the predicted value of the jth scenes, $\overline{s }$ is the average predicted value.

### F-measure

The F-measure presents the positive agreement over the developed prediction system. It presents the weighted harmonic balance between precision and recall measures. It is expressed as follows:

16 $$F{\text{-}}measure=\frac{2*Precision*Recall}{Precision+Recall}.$$

### Memorability testing

Memorability testing is a significant measure employed to define the efficacy of the learning frameworks. It is evaluated by exploring the memory capability of learning parameters. It portrays the proficiency of the prediction system. It is expressed as follows:

17 $${Mem}_{test}= \frac{Count \,\,of \,\,missed\,\, interesting \,\,scenes}{\left(Count \,\,of \,\,interesting \,\,scenes\,\, received-Count \,\,of\,\, interesting \;\;scenes \,\,withdrawn\right)\times 100}.$$

In order to evaluate the performance of the proposed method, an analytic study is performed on the convergence analysis, memory capacity, translational invariance, and losing interest.

### Convergence analysis

The convergence analysis between short-term and online learning modules is done to prove the effectiveness of the results. The proposed Trimmed Q-Learning module looks for the specified target region to learn the next action of the robots. Here, the outcome of the predictor region is not amended due to the dynamic environment. The iteration number decreases rapidly with the trimmed regions during the learning process. Henceforth, the learning parameters converge at the 10th iteration with the lesser epochs. Finally, a maximum of 200 iterations are used for training purposes.

### Memory capacity

The analysis of memory capacity is done to explore the count of trimmed regions used for the learning process. The accuracy of the learning module is an opinionated one. The role of uninteresting objects in the interesting scenes might affect learning ability. Thus, the model is designed to cope with better features and interesting scenes.

### Translational invariance

Here, it is assumed that the results of robot actions are invariant to translations and rotations of both scenes and the action. A set of convoluted operators is used over a spatial action space to generate a Q-function without degrading image quality. It is also not equivariant to all state and action variables. The use of the dynamic trimmed function does efficient memory modules.

### Losing interest

The qualitative test is conducted on the proposed Trimmed Q-learning framework. It is done with the help of the SUN dataset^52^. The objects monitored on the video are relatively stable due to the dynamic background motion. It is intended to test the online learning framework. The use of two critic networks is to define the interestingness level to detect new objects. The detection of similar scenes might drop the interestingness level. Therefore, the hyper parameters related to the action variables are adjusted to the learning parameters.

### Comparative analysis

**Figure 4:**
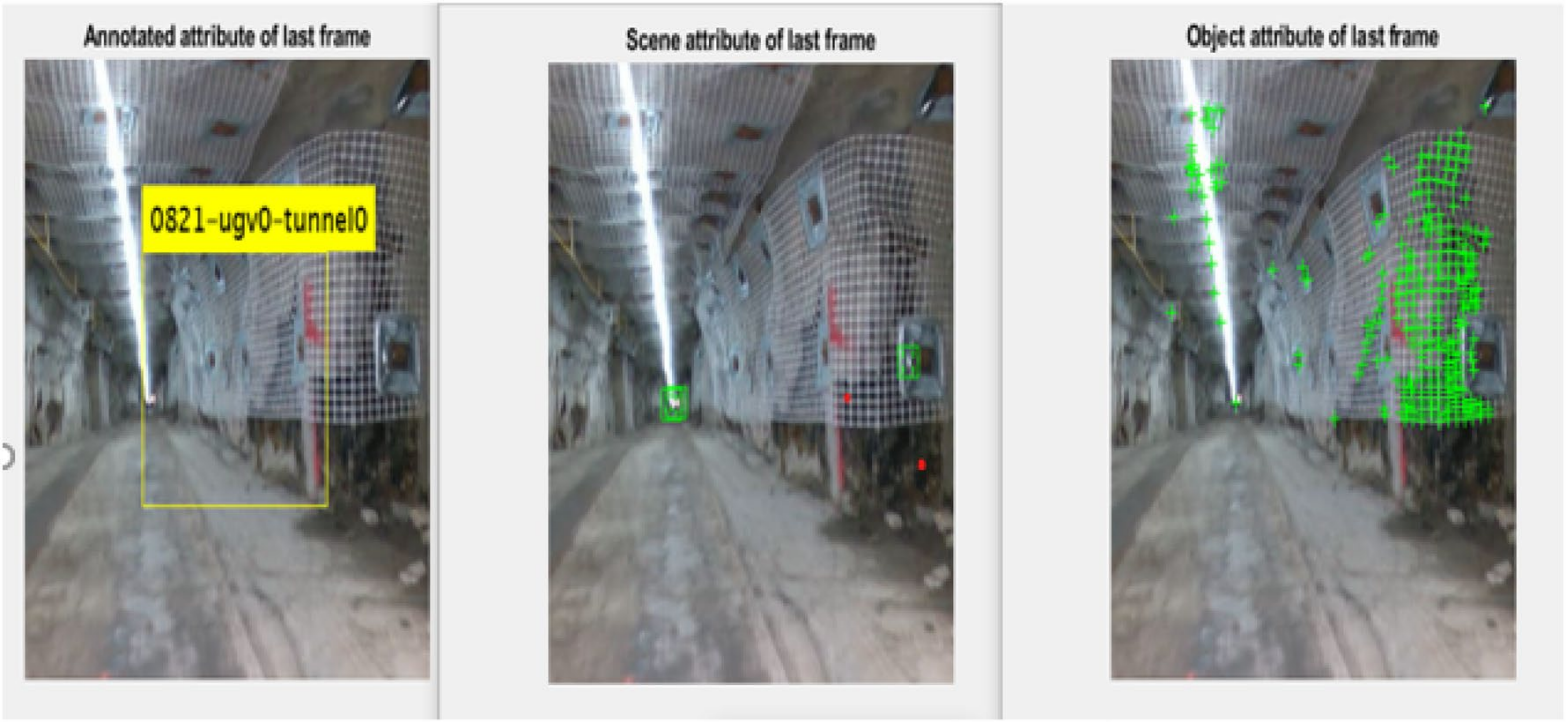
Sample- feature extraction process.

**Figure 5:**
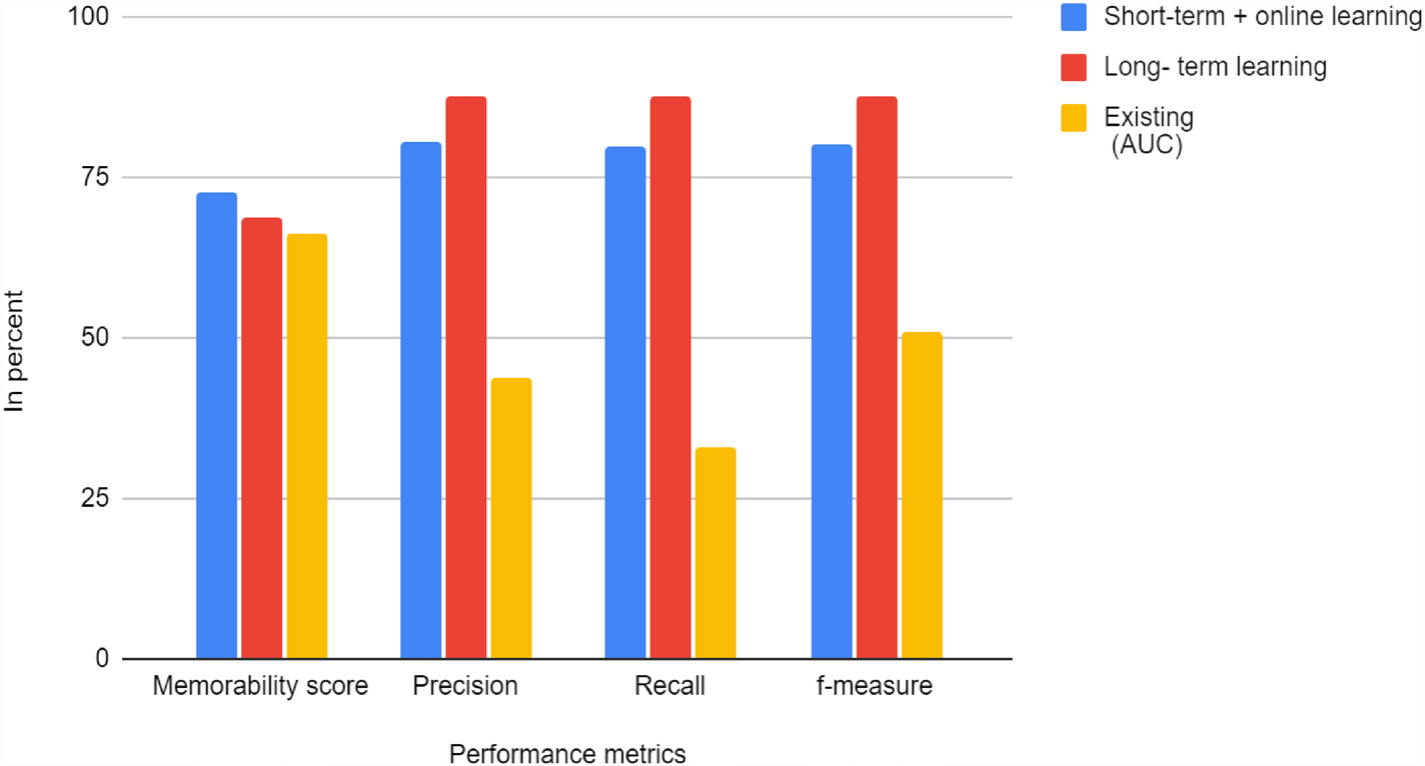
Comparison graph.

The proposed framework outcomes in Table 2 is compared with the existing CNN learning framework^53^. In the previous study, the analysis is carried out in the Area under Curve (AUC). Here, the memorability-based interesting scene-prediction system is introduced. Each frame is described by its count of objects, categories and the intrinsic characteristics. Regardless, many studies reveal several properties with the frames that plays important role in modelling the video memorability. The Fig. 4 presents the feature extraction process and the comparison graph is shown in Fig. 5.

**Table 2:** Proposed framework–outcomes.

Performance metrics	Short-term + online learning	Long- term learning	Existing (AUC)
Memorability score	72.84	68.63	66.2
Precision	80.59	87.62	43.7
Recall	80	87.56	33.0
F-measure	80.29	87.59	50.8

## Conclusion

This paper uses a memorability-based interestingness measure to predict interesting scenes for robotic applications. A novel trimmed Q-learning algorithm is designed to leverage the long-term, short-term and online learning process. Initially, the input videos are modelled into the visual memory schema. Each video frame is accessed by object, scene and annotated attributes. The collected attributes are used to define the roles of a frame that contributed to a long-term learning process. Then, a set of candidate actions with the trimmed regions are explored in a diverse unknown environment contributing to the short-term and online learning process. At last, the interesting scenes with the interesting objects are predicted by estimating the recalling ability. Experiments conducted on public datasets, SubT and SUN databases demonstrate the proposed technique’s efficacy. The proposed framework has yielded a 10−15% better improvement than the existing study.

## Data Availability

The datasets generated or analyzed during the current study are available in the SubT dataset and SUN Dataset repository, https://theairlab.org/dataset/interestingness and https://groups.csail.mit.edu/vision/SUN/hierarchy.html respectively.
